# Identification of novel multi-stage histone deacetylase (HDAC) inhibitors that impair *Schistosoma mansoni* viability and egg production

**DOI:** 10.1186/s13071-018-3268-8

**Published:** 2018-12-27

**Authors:** Alessandra Guidi, Fulvio Saccoccia, Nadia Gennari, Roberto Gimmelli, Emanuela Nizi, Cristiana Lalli, Giacomo Paonessa, Giuliana Papoff, Alberto Bresciani, Giovina Ruberti

**Affiliations:** 1 National Research Council, Institute of Cell Biology and Neurobiology, Campus A. Buzzati-Traverso, Monterotondo (Roma), Italy; 2 Biology Department, IRBM Science Park SpA, Pomezia, Italy; 3 Chemistry Department, IRBM Science Park SpA, Pomezia, Italy

**Keywords:** *Schistosoma mansoni*, HDAC inhibitors, Parasite reproductive systems

## Abstract

**Background:**

Novel anti-schistosomal multi-stage drugs are needed because only a single drug, praziquantel, is available for the treatment of schistosomiasis and is poorly effective on larval and juvenile stages of the parasite. Schistosomes have a complex life-cycle and multiple developmental stages in the intermediate and definitive hosts. Acetylation and deacetylation of histones play pivotal roles in chromatin structure and in the regulation of transcription in eukaryotic cells. Histone deacetylase (HDAC) inhibitors modulate acetylation of several other proteins localized both in the nucleus and in the cytoplasm and therefore impact on many signaling networks and biological processes. Histone post-translational modifications may provide parasites with the ability to readily adapt to changes in gene expression required for their development and adaptation to the host environment. The aim of the present study was to screen a HDAC class I inhibitor library in order to identify and characterize novel multi-stage hit compounds.

**Methods:**

We used a high-throughput assay based on the quantitation of ATP in the *Schistosoma mansoni* larval stage (schistosomula) and screened a library of 1500 class I HDAC inhibitors. Subsequently, a few hits were selected and further characterized by viability assays and phenotypic analyses on adult parasites by carmine red and confocal microscopy.

**Results:**

Three compounds (SmI-124, SmI-148 and SmI-558) that had an effect on the viability of both the schistosomula larval stage and the adult worm were identified. Treatment with sub-lethal doses of SmI-148 and SmI-558 also decreased egg production. Moreover, treatment of adult parasites with SmI-148, and to a lesser extent Sm-124, was associated with histone hyperacetylation. Finally, SmI-148 and SmI-558 treatments of worm pairs caused a phenotype characterized by defects in the parasite reproductive system, with peculiar features in the ovary. In addition, SmI-558 induced oocyte- and vitelline cell-engulfment and signs of degeneration in the uterus and/or oviduct.

**Conclusions:**

We report the screening of a small HDAC inhibitor library and the identification of three novel compounds which impair viability of the *S. mansoni* larval stage and adult pairs. These compounds are useful tools for studying deacetylase activity during parasite development and for interfering with egg production. Characterization of their specificity for selected *S. mansoni versus* human HDAC could provide insights that can be used in optimization and compound design.

## Background

Parasitic diseases cause morbidity and mortality, particularly in the poorest regions of the world. Schistosomiasis is considered the second most important parasitic disease behind malaria in terms of its socio-economic and public health importance, and prevalence in the developing world. It has been estimated that globally over 200 million people are infected with the parasites with a vast majority of them (85%) living in sub-Saharan Africa [1–3]. *Schistosoma mansoni*, *S*. *haematobium* and *S*. *japonicum* are the three most relevant species for human infections [4].

Schistosomes, like other trematodes, display a complex life-cycle which comprises both free-living larvae and parasitic forms with several developmental stages [5]. Throughout its life-cycle, *S. mansoni* must reset its metabolism in order to cope with different living conditions dictated by a variety of environments; drastic changes occur during its development and the transition from cercariae into adult worms. Moreover, whereas most of the trematodes are hermaphrodites, schistosomes are sexually dimorphic and pairing of males and females is required for the maturation of female worms and the production of eggs [6–8]*.* The eggs produced by sexually mature adult females play a key role in both disease transmission after their release in the environment and pathology as they are causing inflammatory processes and granuloma formation in the host tissues leading to organ failure.

Praziquantel (PZQ) is essentially the only drug used for the treatment of schistosomiasis. It is very effective against adult worms of all three *Schistosoma* species [9, 10], but unfortunately, it is poorly active on juvenile and schistosomula immature stages both *in vivo* and *in vitro* [11–14] and does not prevent re-infection [15, 16]. In addition, widespread use of PZQ in both humans and domestic animals, along with the identification of field [17–20] and laboratory isolates [21–24] with reduced susceptibility to PZQ raise serious concerns about the risk of selection of drug-resistance strains. Therefore, new schistomicidal drugs that target multiple stages of the parasite are needed.

Interestingly, it has been suggested that parasites and cancer cells have several properties in common [25]: the ability to survive in the host by hiding and escaping the immune system and the increased metabolic rate activity, due to a higher dependence on lactate fermentation as a preferential energy source, are common features between cancer cells and parasites. It is also well known that tumor cells use epigenetic processes to escape from therapy and immune surveillance [26]. Therefore the epigenome, including DNA methylation and histone modifications, have been thoroughly investigated to identify novel cancer targets in drug discovery programmes [27]. Due to the similarities between cancer cells and parasites, targeting the epigenome has emerged as a new strategy for the treatment of parasitic diseases including schistosomiasis [28–30].

HDACs are the most investigated epigenetic targets in humans and a variety of specific inhibitors, active on cancer cells, have already been discovered [31]. HDAC inhibitors have also been explored in the past years as putative candidate drugs to fight several human parasitic diseases including leishmaniasis, malaria, schistosomiasis, toxoplasmosis and trypanosomiasis [32–34]*.* Importantly, class I HDACs (SmHDAC1, 3 and 8) are expressed in all developmental stages of *Schistosoma*, and *Sm*HDAC8 has been shown to be the most abundant [35]. Moreover, several studies indicate that targeting histone deacetylation activity could represent a promising therapeutic strategy [36–38]*.*

In order to identify new anti-schistosomal molecules, we screened a small library of class I HDAC inhibitors against the larval stage of *S. mansoni* and selected four hits for further characterization on the adult stage of the parasite. Our study identified three novel compounds that impaired viability of both *S. mansoni* larval and adult stages. In addition, when used at sub-lethal doses two compounds, SmI-148 and SmI-558, drastically reduced the number of eggs laid by mature females *in vitro* and induced phenotype alterations in their reproductive system (ovary and oviduct). Finally, analysis of histone acetylation demonstrated the effect on enzymatic activity of two out of four compounds.

## Methods

### Reagents

Dimethyl sulphoxide (DMSO), percoll, Nonidet P-40 (NP40), fetal bovine serum (FBS), thimerosal, gambogic acid, trichostatin A (TSA), bovine serum albumin (BSA), carmine-red and Canada balsam were purchased from Sigma-Aldrich (Saint Lous, USA); CellTiter-Glo (CTG) reagent from Promega (Madison, USA); Dulbecco-Modified Eagle’s Medium (DMEM) with or without phenol red, HEPES, L-glutamine from Lonza (Basel, Switzerland); antibiotic-antimycotic reagent (100×) from Thermo Fisher Scientific (Waltham, USA); HDAC1 enzyme (BMLSE456), substrate (BML-KI104) and developer solution BML-KI105) from Enzo Life Sciences, Inc (Farmingdale, USA); Dacinostat (S1095) from Selleckchem (Munich, Germany); the primary monoclonal anti-α-tubulin antibody (DM1A) from Sigma-Aldrich; the anti-acetylated-lysine (Ac-K2-100) from Cell Signaling Technology (Danvers, USA); goat anti-mouse and anti-rabbit IgG (H+L)-horseradish peroxidase secondary antibodies from Bio-Rad Laboratories (Hercules, USA).

### Collection of HDAC inhibitors

The compound collection used in the present work consisted of approximately 1500 IRBM proprietary compounds with a range of activity against human HDACs. The collection was synthesized in the past two decades as part of multiple drug discovery efforts [39, 40]. Compounds, to be transferred both to biochemical and cell-based assays, were prepared from 10 mM DMSO stock solutions. Mother solutions were serially diluted and transferred to assay plates by an acoustic droplet ejection device (ATS-100, EDC Biosystems, Fremont, USA).

### Human HDAC1 activity assay

The buffer used in the assay was TBS + 1 mM MgCl_2_ + 0.1% BSA. Human HDAC1 was diluted to 1 nM in assay buffer and 15 μl of the diluted enzyme mix were transferred to each well of compound containing microplates (P.N. 4316, Thermo Fisher Scientific). After 10 min incubation at room temperature, 5 μl of assay buffer-diluted substrate (BML-KI104) was added to each well to a final concentration of 80 μM. The reaction was incubated for 1 h at room temperature. To develop the reaction signal, 15 μl of a concentrated (600-fold) assay buffer-diluted developer solution (BML-KI105) was transferred to each well with the addition dacinostat (3 μM final concentration) (S1095) to stop the reaction. Dacinostat (3 μM) was also used as 100% inhibition reference, while DMSO was used as 0% inhibition reference. After 10 min incubation at room temperature the reaction signal was read at 360 nm excitation, 460 nm emission on an EnVision spectrophotometer (PerkinElmer, Waltham, USA).

### HeLa class I HDAC inhibition assay

The buffer used in the assay was TBS + 0.25 mM MgCl_2_ + 0.02% BSA. Human cervical cancer HeLa cells (1 × 10^4^ c/w) were plated in DMEM complete medium with 10% FBS and without phenol red in a 384-well plate and left to recover for 4 h at 37 °C, 5% CO_2_ in a humidified atmosphere. After recovery, compounds were transferred to assay plates as per the compound preparation method. Then, 5 μl of assay buffer-diluted substrate (BML-KI104) were added to each well to a final concentration of 400 μM. The reaction was incubated for 4 h at 37 °C, 5% CO_2_ in a humidified atmosphere. To develop the reaction signal, 15 μl of concentrated assay buffer-diluted developer solution (27-fold) (BML-KI105) were transferred to each well with the addition of 3% NP40 and dacinostat (6 μM final concentration) (S1095) to stop the reaction. Dacinostat (6 μM) was also used as the 100% inhibition reference, while DMSO was used as the 0% inhibition reference. After 10 min incubation at room temperature the reaction signal was read at 360 nm excitation, 460 nm emission on an EnVision spectrophotometer (PerkinElmer).

### Maintenance of the *S. mansoni* life-cycle

As previously described, a Puerto Rican strain of *S*. *mansoni* was maintained by passage through albino *Biomphalaria glabrata*, as the intermediate host, and ICR (CD-1) outbred female mice as the definitive host [41]. Female 4- to 7-week-old mice (Envigo, Udine, Italy) were housed with the following conditions: 22 °C, 65% relative humidity, 12/12 h light/dark photocycle, standard food and water *ad libitum.* Mice were infected with 150–200 single sex or double sex *S. mansoni* cercariae by the tail immersion technique. Adult parasites were harvested from mice 7–8 weeks after infection by reversed perfusion of the hepatic portal system and mesenteric veins.

### Preparation of parasites, viability assays and egg counts

Schistosomula were obtained by mechanical transformation of cercariae using an optimized version of the protocol of Brink et al. [42], previously described by Protasio et al. [43] and adapted in our laboratory [41].

The viability assay on schistosomula (100/well) was carried out as previously described in 384-well, black, tissue culture plates [41]. Briefly, compounds dissolved in DMSO were transferred to wells by using the acoustic droplet ejection technology (ATS-100, EDC Biosystems). DMSO (vehicle) and gambogic acid (10 μM) were used as the negative and positive control in each plate, respectively. Schistosomula were incubated in DMEM medium (without phenol red) with the compounds for 24 h at 37 °C and 5% CO_2_. Next, CellTiter-GLO reagent (CTG) (30 μl) was added and a luminescence signal, proportional to the amount of ATP present in the well, was measured 30 min after CTG addition. The relative luminescence unit (RLU) was obtained by a charge-coupled device (CCD)-based detector (ViewLux, PerkinElmer). The data were analyzed using GraphPad Prism v6.0c software (San Diego, USA). The percentage of dead schistosomula for each compound was calculated as the ATP reduction against vehicle (0%) and gambogic acid (100%).

For studies on adult worms, 5–10 males or couples were incubated with selected compounds in 3–5 ml DMEM complete tissue culture medium for up to 7 days [44]. The compound was given to parasites *in vitro* only once without medium addition and/or replacement.

Survival was monitored daily under a Leica MZ12 stereomicroscope and scores assigned, as previously reported, based on phenotypes [41]. Specifically, we adopted the following phenotype scoring criteria: 3, plate-attached, good movements, clear; 2, slower or diminished movements, darkening, minor tegumental damage; 1, movements heavily lowered, darkening, tegument heavily damaged; 0, dead, lack of any movement. For each sample the total score was determined with the following formula:

$$ \mathrm{Total}\ \mathrm{score}=\frac{\sum \left( Worm\kern0.17em scores\right)}{Number\kern0.17em of\kern0.17em worms} $$

The percentage severity score (viability) was assigned in at least three independent experiments for each compound, relative to vehicle (DMSO). The number of eggs produced by all worm couples *in vitro* was manually counted at day 3 upon compound treatment using an inverted Leica DM IL microscope (Leica Microsystems, Wetzlar, Germany).

### Western blot analysis

Five adult male worms were recovered 24 h after HDAC inhibitor-treatment and washed twice in cold PBS. Parasites were processed by using a modified version of the protocol of Dubois et al. [36]. Briefly, adult worms were resuspended in lysis buffer (200 μl of PBS containing 50 mM HEPES, 150 mM NaCl, 5 mM EGTA, 1% Triton X-100) and sonicated 6 times for 15 s. Next, the samples were centrifuged for 10 min (14,000× *g* at 4 °C), the supernatants removed and the pellets (containing the enriched-nuclear proteins) resuspended in protein loading buffer (100 μl). They were then sonicated 6 times for 15 s, boiled, and analyzed by SDS-PAGE and western blot with anti-α-tubulin DM1A (1:5000) or anti-acetylated-lysine (1:4000) following the antibody manufacturer’s instructions. A Chemidoc XRS (Bio-Rad) with a chemi-luminescent camera and ImageLab 4.0 software were used for the acquisition of images. Each western blot analysis was calibrated by ponceau-staining.

### Carmine-red staining and confocal laser scanning microscopy analysis

Carmine-red staining was performed as previously described [44]. Images were taken on an Olympus FV1200 confocal laser-scanning microscope using an UPlanFLN 40× immersion oil objective (NA = 1.30) with optical pinhole at 1 AU and a multiline argon laser at 488 nm as the excitation source. The images were collected as a single stack.

### Statistical analysis

All statistical tests were performed using GraphPad Prism v.6.0c software. All viability data are shown as the mean ± standard error of the mean (SEM) or ± standard deviation (SD) as indicated. Differences in the viability scores were analyzed by Student’s t-test. For all experiments, *P*-values < 0.05 were considered to be statistically significant.

## Results and discussion

### Identification of compounds able to kill schistosomula from a HDAC inhibitor collection

**Fig. 1:**
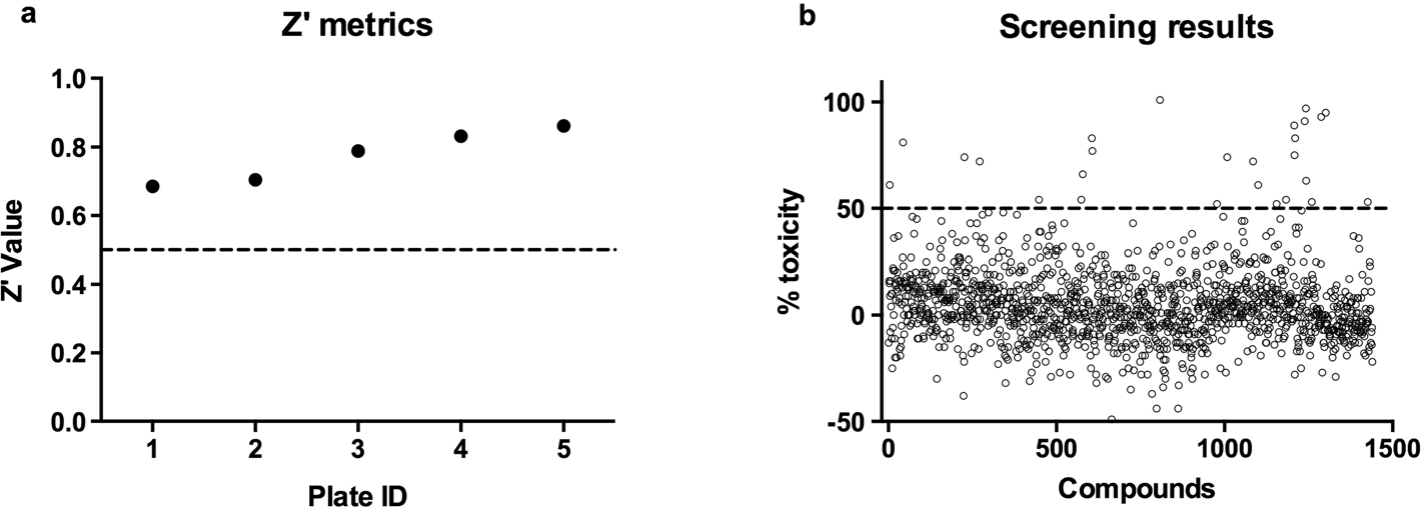
HDAC inhibitors collection screening. **a** For each microplate the quality of the assay was carried out by the Z’ calculation. Positive controls wells (*n* = 16) were treated with 10 μM gambogic acid whereas negative wells (*n* = 16) were treated with vehicle (DMSO). All plates were found to be above the 0.5 quality threshold. **b** The percentage of ATP reduction (death of schistosomula) for all compounds (tested at 10 μM) normalized against DMSO and gambogic acid

A compound collection comprising approximately 1500 molecules was screened at a single concentration of 10 μM using a previously described schistosomula viability assay [41]*.* The quality of the screening was assessed by the Z’ value [45], calculated for each tested plate using vehicle (DMSO) and gambogic acid dispensed wells as negative and positive controls, respectively. The Z’ was above the 0.5 threshold in all plates, the lowest acceptable value for a robust assay (Fig. 1a). The percentage of dead schistosomula for each compound was calculated as the ATP reduction against vehicle (0%) and gambogic acid (100%). The positivity threshold was arbitrarily set to 50% (Fig. 1b). By using this threshold, 27 active compounds were identified (1.6% hit rate). The active compounds were quality-controlled by LC-MS and re-tested in the schistosomula viability assay at three different concentrations (50, 10 and 2 μM) for confirmation. The activity of 10 compounds was not confirmed and an additional 10 compounds consisted of < 90% impurities. Four compounds of the remaining seven were identified as prototype molecules for further profiling.

**Fig. 2:**
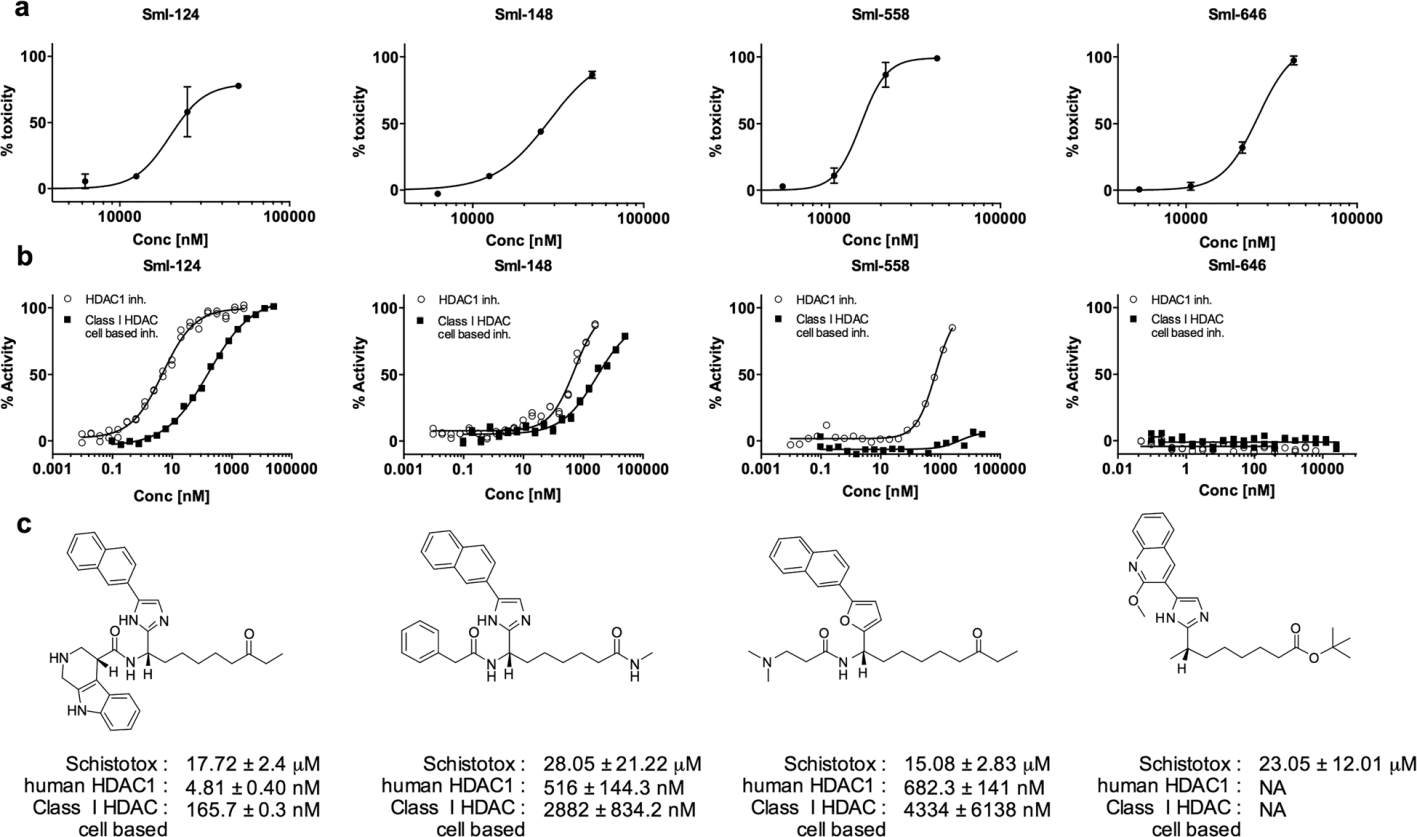
Activity of selected hit compounds. **a** Dose response curves of the hit compounds on schistosomula. The y-axis indicates the percentage of ATP reduction (death of schistosomula) normalized against DMSO (0%) and gambogic acid 10 μM (100%). Each point represents the average and standard deviation of three independent experiments. **b** Inhibitory dose response curves of the hit compounds on human HDAC1 and on cellular deacetylase activity (class I HDAC cell based, on HeLa cells). Each point represents the average and standard deviation of three independent experiments. **c** Chemical structures of hit compounds and activity summary. *Abbreviation*: NA, not active

Next, the selected compounds were tested in a dose-response fashion in the schistosomula viability assay: all had potencies in the range of 10–20 μM (Fig. 2a). Furthermore, they were assayed for their activity on human recombinant HDAC1 and on a cell-based assay, which measures class I HDAC inhibition in human HeLa cells, and the results showed a different sensitivity to the compounds (Fig. 2b). In particular, for both assays SmI-124 exhibited very good activity whilst SmI-148 and SmI-646 showed modest or absent activity, respectively. In addition, SmI-558 was apparently not active against class I HDACs in cells. This finding suggests that there is the potential to gain specificity for *S. mansoni* HDACs with respect to human ones. The structural features of the compounds tested, shown in Fig. 2c, is the presence of different Zn-binding groups (ketone, carboxylate esters and secondary amide) in the terminal position of an alkyl chain linked to a five membered heterocycle having a biaryl substituent (naphthyl or quinolone).

### Compound-treatment affects *S. mansoni* adult parasite viability and egg production *in vitro*

**Fig. 3:**
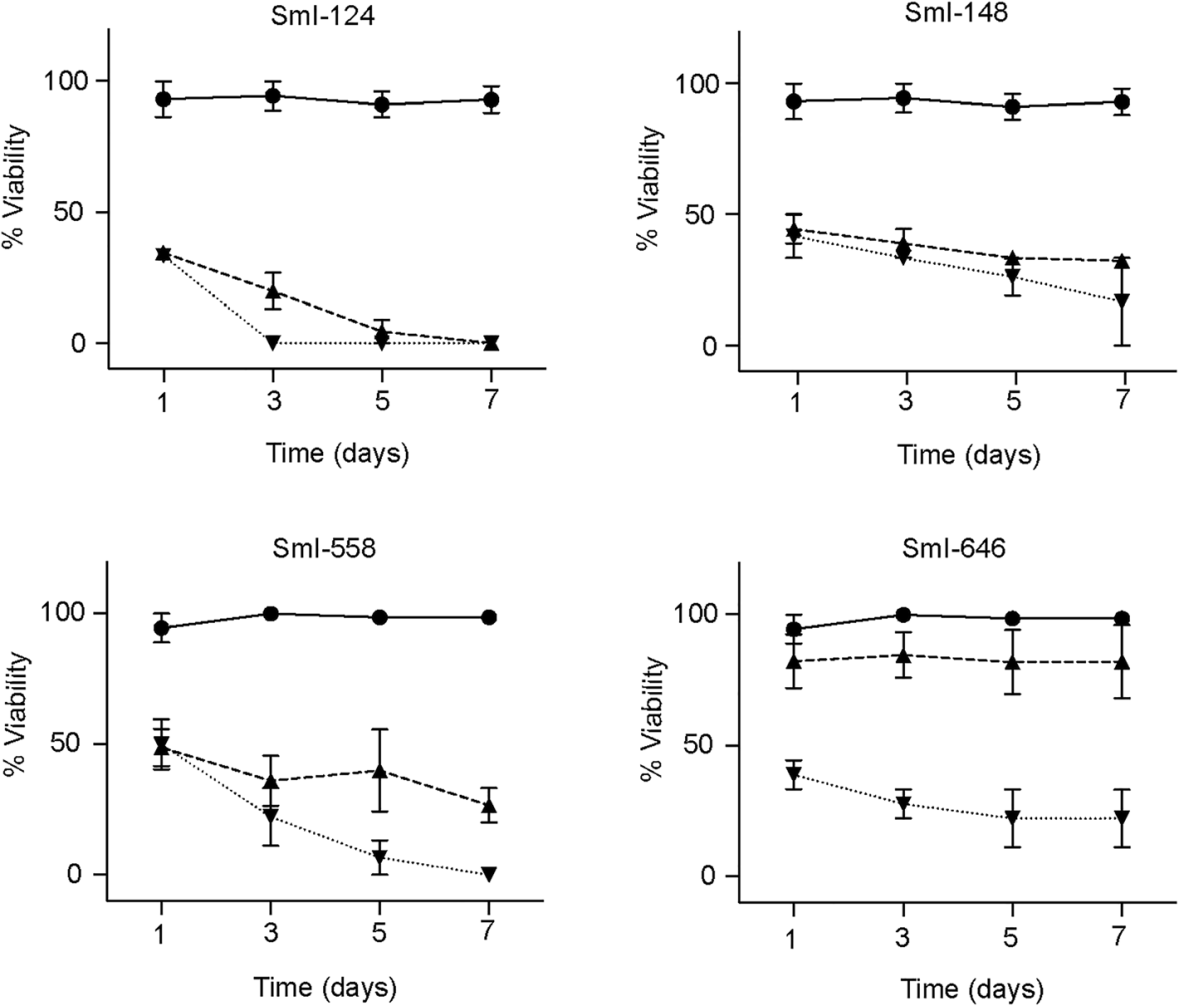
Activity of selected hit compounds on adult *S. mansoni* worms. The percentage of viability of adult male worms, exposed to the indicated compounds at the concentration of 10 μM (triangle) and 20 μM (inverted triangle), scored at different time points (x-axis). DMSO (vehicle, circle) represents the negative control. Each point represents the average ± SEM of three independent experiments

**Fig. 4:**
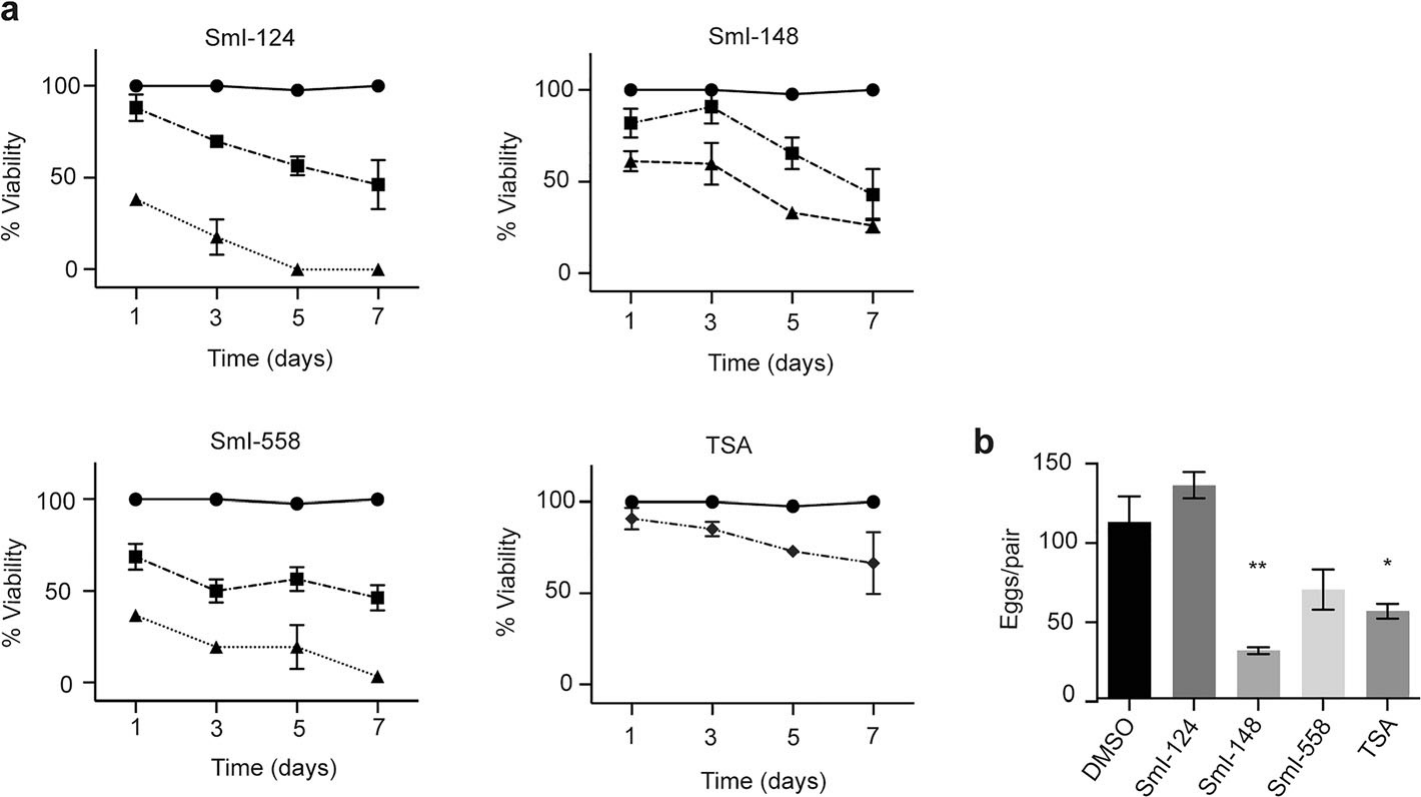
Effects of selected hit compounds on *S. mansoni* adult worm pairs viability and egg production *in vitro.* **a** Viability curves of adult worm pairs treated with SmI-124, SmI-148, and SmI-558 at 5 (square) or 10 μM (triangle) concentrations. DMSO (circle) and trichostatin A (TSA) (diamond) treatments represent the negative and positive controls, respectively. **b** Total egg counts laid by pairs treated with a sub-lethal dose (5 μM) of the indicated compounds normalized to worm couples are shown. The mean data ± SEM of three independent experiments are shown. The levels of statistical significance are indicated above bars; *P < 0.05, **P < 0.01, Student’s t-test

The efficacy of the hit compounds, selected in the schistosomula screening, was also assessed by survival assays and bright-field microscopy analyses on adult male worms. Parasites were subjected to treatment with the compounds (10 and 20 μM) and checked daily for any morphological or lethal alteration. As shown in Fig. 3, three out of four compounds (SmI-124, SmI-148 and SmI-558) led to a strong decrease in viability within seven days of compound exposure using both concentrations. These compounds were then tested against mature pairs using to monitor the effect on viability for both male and female worms as well as to detect any differences on egg laying (Fig. 4a). We found that all three compounds exhibited a lethal action when used at the highest concentration (10 μM) but not at the lowest one (5 μM). Moreover a strong reduction in the number of eggs laid by worm pairs treated with 5 μM SmI-148 and SmI-558 three days after treatment was recorded (Fig. 4b). In addition, as SmI-558 led to a decreased viability of parasites (about 50%) three days after the treatment with 5 μM, pairs were also assayed with lower concentrations of the compound. Therefore, using SmI-558 at 2.5 μM concentration worms appeared fully viable but the number of eggs laid *in vitro* by female worms did not differ from that of control samples. The effects of SmI-148 and SmI-558 on both schistosomula and mature worms viability, along with their negative impact on egg laying, are of particular interest for the development of anti-schistosomal compounds.

### The HDAC inhibitors SmI-124 and SmI-148 induce histone hyperacetylation in adult worms

**Fig. 5:**
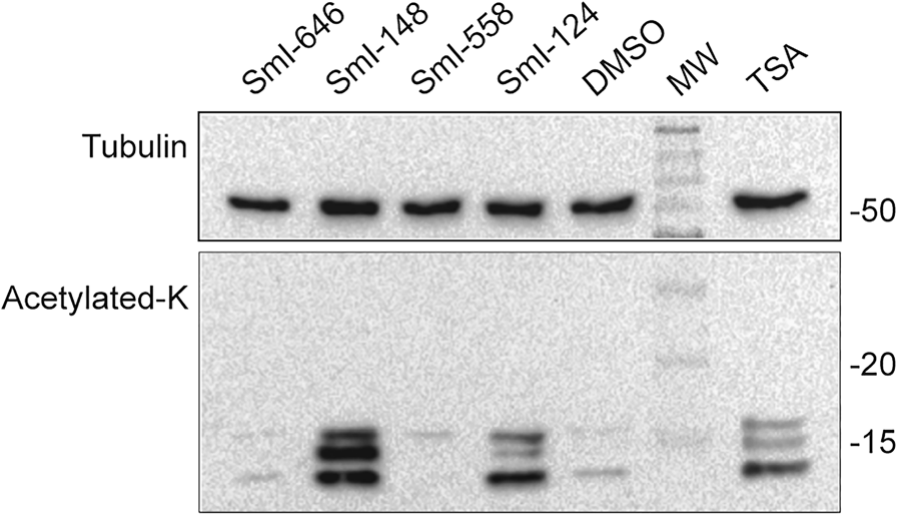
Histone acetylation analysis in HDAC inhibitors-treated adult *S. mansoni* parasites. Representative immunoblots of the histone-enriched protein fractions extracted from *S. mansoni* adult worms incubated with anti-acetylated lysine antibody (acetylated-K) or anti-tubulin, as a sample loading control. Worms have been treated for 24 h with 1 μM of the HDAC pan-inhibitor, TSA or with the indicated HDAC inhibitors (10 μM) or with DMSO (vehicle) used as control. Lane MW: molecular weight marker size. The western blot is representative of 3 independent experiments

In order to investigate the activity of the selected compounds on HDAC, the levels of histone acetylation were investigated 24 hours after exposure of parasites to the inhibitors (10 μM) by immunoblot of nuclear-enriched cell fractions. Our results indicate that SmI-148- and SmI-124-treated worm lysates showed histone hyperacetylation comparable to the levels found in the pan-inhibitor TSA-treated worms with the strongest effect exerted by the first compound (Fig. 5). Three distinct bands ranging from ~11 to ~16 kDa were detected: the lower mobility bands likely correspond to hyperacetylated forms of H2B (~13 kDa) and H2A (~16 kDa) [46] whereas the one migrating faster is ascribable to histone H4 (11 kDa). It is interesting to note that the strongest hyperacetylation signal was in histone H2 which along with H3 and H4 are considered the core histones.

HDACs target histones and also other nuclear and cytoplasmic proteins such as cytoskeletal proteins, chaperones, transcription factors, proteins involved in chromatin remodeling, and signaling mediators. They are therefore also named lysine deacetylases [47]. Indeed, proteome-wide lysine acetylation in 42-days-old *S. japonicum* worms identified approximately 1000 acetylated proteins [48]. Furthermore, a comparative analysis of 18- and 28-days-old *S. japonicum* worm acetylation profiles indicated that acetylation modulated several biological functions during parasite development, including gene transcription and translation, glycometabolism and lipid metabolism, muscular movement and protein degradation [49]. Therefore, we cannot exclude that SmI-558, as well as SmI-124 and SmI-148, target non-histone proteins. This aspect remains to be investigated.

### SmI-148 and SmI-558 treatments cause alterations of parasite reproductive systems

**Fig. 6:**
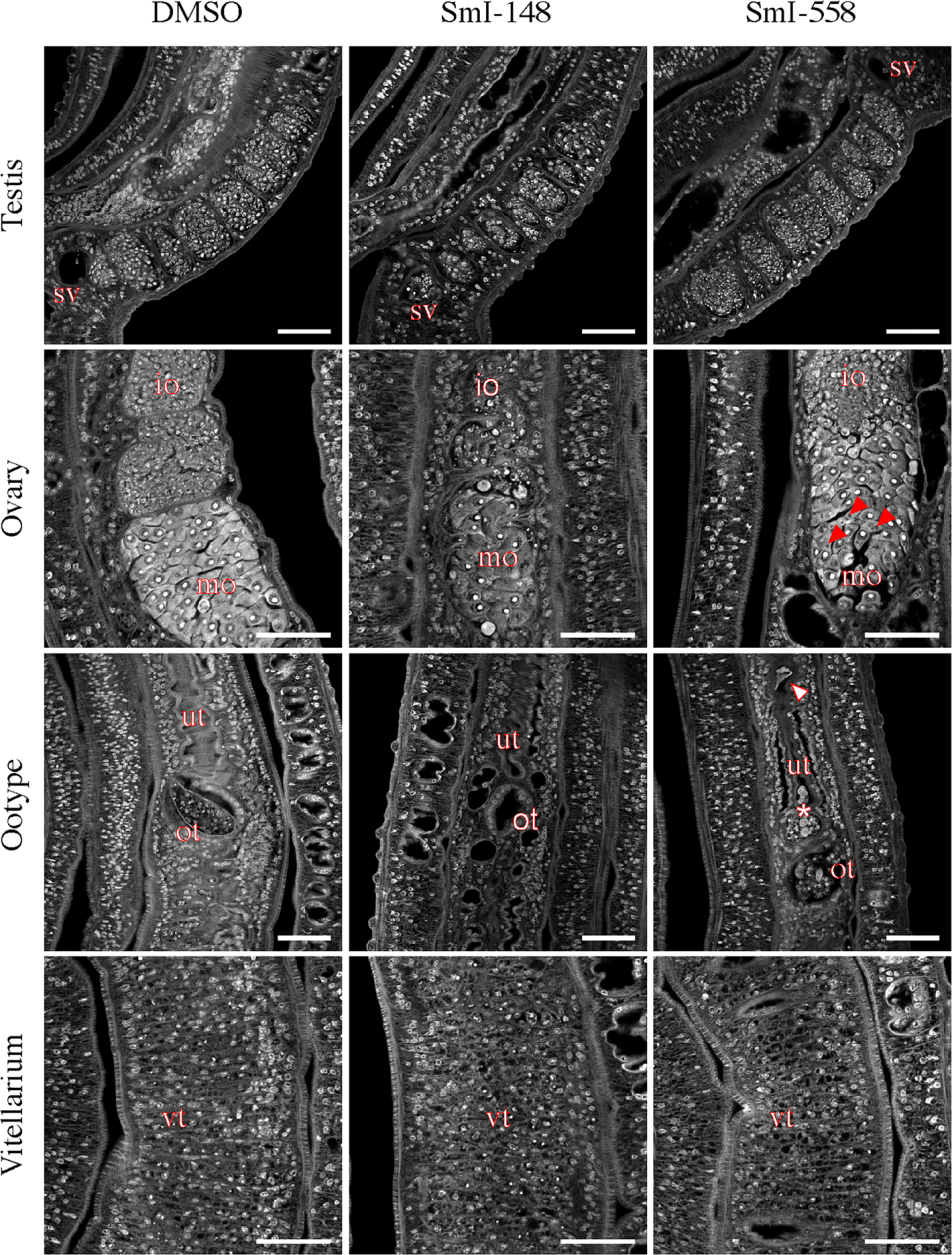
Effects of selected HDAC inhibitors on the reproductive systems of adult *S. mansoni* pairs. Representative confocal scanning laser images of adult *S. mansoni* pairs treated for 72 h with DMSO (vehicle), SmI-148 at 5 μM, SmI-558 at 2.5 μM (testis, ovary and ootype) or 5 μM (vitellarium) and stained with carmine red. *Abbreviations*: sv, seminal vescicle; io, immature oocyte; mo, mature oocyte; ut, uterus; ot, ootype; vt, vitellarium. Red arrows indicate black spots in mature oocyte, white arrow indicates a deformed egg in uterus, and asterisk indicates cells accumulated in the first part of the uterus. *Scale-bars*: 50 μm

**Fig. 7:**
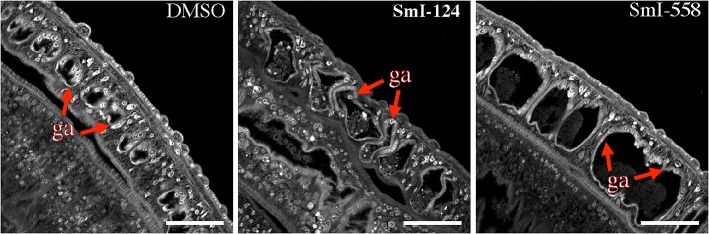
Effects of selected HDAC inhibitors on adult *S. mansoni* gut. Representative confocal scanning laser images of adult *S. mansoni* males treated for 72 h with SmI-558 and SmI-124 (5 μM) and stained with carmine red. In the figure gastrodermis (ga) is indicated. *Scale-bars*: 50 μm

The phenotypic alterations, induced by the selected HDAC inhibitors, were also investigated by carmine-red staining and confocal laser scanning microscopy analysis. Worm pairs treated with SmI-148 showed notable morphological alterations in both testes and ovaries. Specifically, a decrease of cellularity in testes lobes and marked morphological changes in the ovaries were observed (Fig. 6). In particular, the ovaries of SmI-148-treated samples appeared smaller with a decrease in the numbers of both immature and mature oocytes compared to controls (Fig. 6). In addition, the occurrence of eggs in the ootype and uterus was rare and eggs when present were not properly formed. The sub-lethal dose (2.5 μM) of SmI-558, that did not impact egg production *in vitro* (Fig. 4), induced however morphological alterations in the female reproductive organs. The ovaries of SmI-558-treated parasites preserved their overall morphology, but displayed oocytes (both immature and mature) with a wide range of degeneration including black spots in the mature oocytes (Fig. 6). Moreover, the uterus showed dysplastic eggs and dispersed cells and the ootype presented disorganized oocytes and vitelline cells (Fig. 6). The female worms of pairs treated with SmI-148 or SmI-558 at 5 μM also showed alterations in the vitellarium, a decreased cellularity in vitelline follicles and an increase in black-stained cavities (Fig. 6). In contrast, treatment of worm couples with SmI-124 did not cause major alterations in the male and female reproductive systems: gut dilation with detachment of the gastrodermis and accumulation of particle aggregates in the lumen was the main alteration observed. Gut dilatation was also observed in parasites treated with SmI-558 (Fig. 7).

Overall these results highlight an effect of HDAC inhibitors SmI-148 and SmI-558 on the female reproductive system that is consistent with their detrimental effect on egg production.

## Conclusions

In summary, we successfully screened a library of HDAC inhibitors on *S. mansoni* larvae (schistosomula) and further characterized four selected hits on adult parasites. Three of them (SmI-124, SmI-148 and SmI-558) were active on both larval and adult stages with SmI-148 and SmI-558 impairing both egg production and causing morphological alterations of the parasite reproductive systems. Importantly, SmI-148 showed a modest activity on human recombinant HDAC1 and a class I HDACs assay in HeLa cells and SmI-558 was apparently not active against class I HDACs in cells. Further profiling of both compounds against selected human and *S. mansoni* HDACs, including *Sm*HDAC8, could lead to the development of parasite-specific HDAC inhibitors to be further characterized also in murine models of schistosomiasis.

## Abbreviations

HDAC: histone deacetylase
PZQ: praziquantel
TSA: trichostatin A
DMSO: dimethyl sulphoxide
NP40: Nonidet P-40
FBS: fetal bovine serum
BSA: bovine serum albumin
CTG: CellTiter-Glo
*Sm*: *Schistosoma mansoni*
